# Expression and functional characterization of the TRPV3 channel in domestic cat (*Felis catus*) mature oocytes

**DOI:** 10.1186/s40659-026-00709-7

**Published:** 2026-07-06

**Authors:** Sebastián Vergara, José Duarte-Zúñiga, Claudio Hidalgo, Constanza Suárez-Suárez, Karen Castillo, Matthias Piesche, Felipe Aguilera, Rafael A. Fissore, Fernando Hinostroza, Daniel Veraguas-Dávila, Ingrid Carvacho

**Affiliations:** 1 Laboratorio de Canales Iónicos y Reproducción, Departamento de Medicina Traslacional, Facultad de Medicina, Universidad Católica del Maule, Talca, Chile; 2 Escuela de Ingeniería en Biotecnología, Facultad de Ciencias Agrarias y Forestales, Universidad Católica del Maule, Talca, Chile; 3 Instituto de Neurociencia, Centro Interdisciplinario de Neurociencia de Valparaíso (CINV), Facultad de Ciencias, Universidad de Valparaíso, Valparaíso, Chile; 4 Laboratorio de Investigaciones Biomédicas, Centro Oncológico, Facultad de Medicina, Universidad Católica del Maule, Talca, Chile; 5 Departamento de Bioquímica y Biología Molecular, Facultad de Ciencias Biológicas, Universidad de Concepción, Concepción, Chile; 6 Department of Veterinary and Animal Sciences, University of Massachusetts Amherst, Amherst, MA USA; 7 Laboratorio de Neuromodulación, Centro de Investigación de Estudios Avanzados del Maule, Vicerrectoría de Investigación y Postgrado, Universidad Católica del Maule, Talca, Chile; 8 Centro de Investigación en Neuropsicología y Neurociencias Cognitivas, Facultad de Ciencias de la Salud, Universidad Católica del Maule, Talca, Chile; 9 Centro para la Investigación Traslacional en Neurofarmacología, Universidad de Valparaíso, Valparaíso, Chile; 10 Departamento de Ciencia Animal, Facultad de Ciencias Veterinarias y Pecuarias, Universidad de Chile, Santiago, Chile

**Keywords:** Cat oocytes, TRPV3 channel, Oocyte physiology, 2-APB binding sites

## Abstract

**Background:**

Intracellular calcium (Ca^2+^) signaling is essential for oocyte maturation, activation, and fertilization, with repetitive Ca^2+^ transients elicited at fertilization being critical for egg activation and early embryonic development. Mouse oocytes express several non-selective cation channels to support these oscillations, including TRPV3, a member of the transient receptor potential (TRP) channel family. In addition to Ca^2+^, TRPV3 mediates zinc (Zn^2+^) influx, which is a modulator of cortical granules (CGs) distribution and actin organization. In mammals, CG exocytosis mediates the fertilization-induced block to polyspermy, and pharmacological activation of TRPV3 in mouse oocytes elicits Ca^2+^ influx sufficient to trigger activation and parthenogenesis. Despite these critical roles in mice, the expression and function of TRPV3 in other mammals remain unexplored. Here, we evaluate the functional expression of TRPV3 channels in cat oocytes.

**Methods:**

Ovaries from domestic cats were obtained during ovariohysterectomies, and oocytes were isolated and matured *in vitro*. *Trpv3* expression was assessed by RT-PCR from ovaries and germinal vesicle (GV) and metaphase II (MII) oocytes, while TRPV3 localization was evaluated by immunocytochemistry. Mouse WT and TRPV3-knockout oocytes were used as controls for antibody specificity. Functional channel activity was examined using Ca^2+^ imaging following addition of the TRPV3 agonist 2-APB. Three-dimensional modelling, molecular docking, and comparative sequence analysis of feline, mouse, and human TRPV3 proteins were aligned with MAFFT, focusing on identical and biochemically similar residues within the 2-APB-binding sites as well as pore-forming and temperature-sensing domains to assess potential species-specific functional differences.

**Results:**

We detected *Trpv3* transcripts in cat ovaries and in GV and MII oocytes. Immunocytochemistry confirmed TRPV3 protein localization at the oocyte membrane in cats, consistent with reports in mouse oocytes. Additionally, addition of 2-APB elicited robust intracellular increase in Ca^2+^ in MII cat eggs, demonstrating functional TRPV3 channel activity. Comparative analyses revealed non-conservative substitutions in feline TRPV3 compared to mouse and human TRPV3, particularly within the pore-forming, channel gating, and temperature sensing regions, offering a molecular explanation for species-specific differences in TRPV3 function.

**Conclusions:**

Our results demonstrate functional TRPV3 expression in domestic cat oocytes. We find distinctive features in feline TRPV3 compared to rodent and human orthologs. These insights support the development of tailored artificial oocyte activation protocols in cats, with potential applications to Assisted Reproductive Technologies (ART) for endangered felids.

**Supplementary Information:**

The online version contains supplementary material available at https://doi.org/10.1186/s40659-026-00709-7.

## Background

The conservation of endangered wild felids represents a critical challenge in wildlife biology, as nearly half of all felid species worldwide are currently threatened with extinction [1]. According to the International Union for Conservation of Nature (IUCN), 14 felid species are currently classified as vulnerable and four as endangered (IUCN, 2025). The domestic cat (*Felis silvestris catus*) is widely used as a biomedical and reproductive model for developing assisted reproductive technologies (ARTs) in endangered felids [2]. In Chile, five native felid species inhabit the territory, among which the guiña or kodkod (*Leopardus guigna*) is of particular conservation concern. This species is listed as vulnerable by the IUCN due to its small and declining population, one of the most restricted geographic distributions among felids, and an estimated number of fewer than 100,000 reproductive-active adults [3]. Habitat fragmentation resulting from human activity, combined with direct persecution, is a major driver of this decline [4]. Preserving the genetic diversity of such endangered felids required innovative reproductive approaches to complement *in situ* conservation efforts.

ARTs, artificial insemination (AI), and *in vitro* embryo production (IVP) have become valuable *ex situ* tools for preserving the genetic diversity of endangered felids. IVP techniques, such as *in vitro* fertilization (IVF), intracytoplasmic sperm injection (ICSI), and interspecific somatic cell nuclear transfer (iSCNT), have successfully yielded live offspring in domestic cats and several endangered wild felid species [5, 6]. A notable example is the use of iSCNT, where enucleated domestic cat oocytes serves as recipients for nuclei from endangered felids. This approach has successfully produced live cloned offspring of the African wildcat (*Felis lybica*) and the sand cat (*Felis margarita*) [7, 8]. By leveraging the domestic cat as an oocyte donor, iSCNT circumvents the need to obtain gametes directly from endangered felids. Recent studies demonstrated that early stage kodkod embryos can be generated through iSCNT [9], and that kodkod oocytes can be matured in vitro and parthenogenetically activated to develop into blastocysts [10]. These approaches represents a potential alternative for preserving genetic diversity, given the challenges associated with gamete collection in this protected species. However, felid embryos generated by iSCNT often exhibit low developmental rates, poor blastocyst formation, and reduced implantation efficiency [11], partly due to the limited understanding of oocyte physiology and the fertilization signaling cascades in felines, highlighting the need for species-specific refinements of ART protocols. During fertilization, sperm-oocyte fusion triggers a signal transduction cascade, known as “egg activation”, that converts the oocyte into a diploid zygote, initiating embryo development [12]. Egg activation is triggered by the release of the sperm-specific phospholipase C ζ into the oocyte, and it involves a series of cellular processes that mostly depend on repeated increases in intracellular calcium (Ca^2+^) [13]. Importantly, Ca^2+^ influx is essential for both oocyte maturation and to support the oscillations of fertilization in mammals [12]. Moreover, an intracellular Ca^2+^ signal is the first signal of fertilization in all the species studied up to date [14]. Consequently, several experimental strategies have been developed to increase intracellular Ca^2+^ to activate eggs [15]. However, a limited number of studies have assessed the suitability of activation protocols for domestic cat oocytes, and most existing protocols were extrapolated from murine and bovine models [16, 20], yielding only modest success in felids. Contributing factors likely include an incomplete understanding of the molecular mechanisms that maintain metaphase II (MII) arrest and regulate fertilization in cats, as well as the reported frequent spontaneous activation of cat oocytes observed after *in vitro* maturation (IVM) and *in vitro* culture (IVC) [18]. These results emphasize the need to elucidate the signal pathway that sustains MII arrest and the Ca^2+^ influx channels that operate in feline gametes.

Transient Receptor Potential (TRP) channels are a family of non-selective cation channels that serve as cellular sensors for diverse physicochemical cues like temperature, pH, and osmolarity [21]. One family member, the TRP Vanilloid 3 (TRPV3) channel, is robustly and differentially expressed in mouse oocytes: its function increases during maturation and peaks at the MII stage [22]. Pharmacological TRPV3 activation in MII eggs induces robust Ca^2+^ entry sufficient to trigger egg activation and parthenogenesis (22). Consistently, TRPV3 also mediates strontium (Sr^2+^) influx, noteworthy because Sr^2+^ is a widely used common parthenogenetic agent, including in cats [16, 19, 23, 25]. These findings suggest that domestic cat oocytes may possess a functional TRPV3-like and/or TRPV3 channel, but its expression and functional activation remain unconfirmed in this species. In addition, beyond Ca^2+^, TRPV3 has recently been suggested in regulating zinc (Zn^2+^) dynamics in eggs, since TRPV3-null mouse eggs exhibit altered Zn^2+^ influx and concentration, leading to reduced egg developmental potential underscoring the broader significance of this channel in oocyte physiology [26]. Elucidating the function of TRPV3 in domestic cat oocytes could enhance the efficiency of *in vitro* maturation (IVM) and support the development of species-specific activation protocols for applications such as iSCNT.

This study aimed to characterize TRPV3 expression in germinal vesicle (GV) and metaphase MII oocytes (eggs) of domestic cats. To accomplish this, we combined molecular and functional approaches, including transcript and protein analysis to detect TRPV3 in cat oocytes, and live-cell Ca^2+^-imaging to evaluate TRPV3- mediated Ca^2+^ influx. We performed comparative sequence analyses to place feline TRPV3 structure and potential function in the context of mouse and human orthologs. Our results demonstrate that domestic cat oocytes express TRPV3 transcripts and protein, with TRPV3 localized in the plasma membrane.

## Methods

### Collection and *in vitro* maturation of cat oocytes

A total of 62 ovaries from 31 domestic cats (*Felis catus*, 4 months to 4 years old) were collected during routine ovariohysterectomies (OVH) performed in local veterinary clinics for clinical or reproductive control purposes and transported to the laboratory within 6 h of excision, where the oocyte collection was performed.

Germinal Vesicle (GV) oocytes were obtained by mechanically dissecting ovarian tissue in a handling medium consisting of M199 (Sigma, Cat. M4530) supplemented with 20 mM HEPES (Sigma, Cat. H3375) and 10% fetal FBS, pH 7.4. Metaphase II (MII) eggs were obtained by culturing GV-stage oocytes in maturation medium composed of M199 supplemented with 4 mM NaHCO_3_, 10 μg/ml estradiol (E_2_), 20 ng/ml epidermal growth factor (EGF), 10 μl/ml ITS, 0.5 IU/ml FSH, 1.0 IU/ml LH, 1.0 mM glutamine, 0.36 mM sodium pyruvate, 2.2 mM calcium lactate, and 0.3% BSA. Oocytes were incubated for 24 h at 38.5 °C in a humidified atmosphere containing 5% CO_2_ [27]. Cumulus cells were removed by pipetting in the presence of 1 mg/ml hyaluronidase. For zona pellucida (ZP) removal, oocytes were initially exposed to 2 mg/ml pronase, followed by brief immersion in acid Tyrode’s solution until the ZP began to contract. Once shrinkage occurred, oocytes were immediately transferred to the handling medium, allowing the zona to re-expand and be removed easily. MII stage of the oocytes was assessed by visual confirmation of first polar body extrusion.

### Superovulation and isolation of mouse eggs

Metaphase II (MII) oocytes (eggs) were isolated from 6- to 16-week-old wild-type (WT) and *Trpv3* knockout (TRPV3KO, V3KO) female mice following standard hormonal stimulation. Mice received an intraperitoneal (i.p.) injection of 5 IU of pregnant mare serum gonadotropin (PMSG), followed 48 h later by 5 IU of human chorionic gonadotropin (hCG). Ovulated MII-arrested eggs were collected from the oviductal ampullae 13–15 h after hCG administration and maintained in HEPES-buffered Tyrode lactate (TL-HEPES) supplemented with 5% fetal bovine serum (FBS). Cumulus cells were removed by incubating the samples in TL-HEPES containing 2.6 mg/ml bovine testicular hyaluronidase (Sigma, H3506), followed by gentle pipetting.

### Oocyte diameter and * zona pellucida *thickness measurements

Oocyte diameter measurement was performed using the ImageJ2 v1.54p software v.X (NIH), by tracing a line in the equatorial region of the oocyte. The mean cytoplasmic diameter of mouse oocytes was assessed using a Mann–Whitney test. The *zona pellucida* (ZP) thickness was measured by tracing a line through the ZP using ImageJ2 v1.54p (NIH); the oocyte was not included in the measurement. ZP thickness was determined by an unpaired t-test with Welch’s correction. A total of 21 mouse oocytes and 16 cat oocytes were used for both analyses.

### TRPV3 Immunocytochemistry in mouse and cat oocytes

Immunocytochemistry was performed in WT and TRPV3KO mouse eggs based on the Neuromab *Immunofluorescence protocol of Cultured Cells protocol* with the modifications as detailed below. Isolated MII-arrested eggs were fixed in 4% paraformaldehyde (Electron Microscopy Sciences, 15,710) for 20 min at room temperature (RT) under gentle agitation. The zona pellucida (ZP) was removed using Tyrode´s acid solution (pH 2.5), followed by three washes in PBS-T (150 mM NaCl and 0.1% Triton X-100 in 10 mM phosphate buffer, pH 7.4).

Samples were blocked in 4% non-fat dry milk prepared in TBS-T (150 mM NaCl and 0.1% Triton X-100 in 20 mM Tris–HCl, pH 8.0, “blocking solution”) for 1 h at RT with gentle agitation. Eggs were then incubated with anti-TRPV3 primary antibody (Neuromab, clone N15/4, 75–043, 10 µg/ml), diluted in blocking solution, for 1 h 30 min at RT. Excess antibody was removed by three washes (5 min each) in blocking solution.

Next, oocytes were incubated with Alexa Fluor 488-conjugated secondary antibody (Invitrogen, A32723), diluted in blocking solution, for 1 h at RT in the dark. After incubation, eggs were washed in PBS-T, protected from light.

Samples were mounted in µ-Slide 18 Well Glass Bottom chambers (Ibidi, Cat. No. 81817) using 100 µl of 1X PBS. Images were acquired using a Nikon AXR + NSPARC Confocal Laser Microscope System at the Institute for Applied Life Sciences, University of Massachusetts Amherst.

Following ZP removal, cat GV, and in vitro-matured MII oocytes were processed for immunocytochemistry using the protocol described for mouse oocytes above, unless otherwise indicated. Oocytes were fixed in 4% paraformaldehyde (Sigma-Aldrich, P6148) prepared in PBS containing 0.1% BSA for 20 min at RT under gentle agitation, followed by three washes in PBS-T. Blocking was performed for 1 h at RT with agitation.

GV and MII oocytes were incubated with the anti-TRPV3 primary antibody (20 µg/ml), diluted in blocking solution for 2 h at RT under gentle agitation. After three washes, samples were incubated with Alexa Fluor 488-conjugated secondary antibody (Abcam, ab150105; 1:500 dilution) and rhodamine phalloidin (Cytoskeleton Inc., Cat. #PHDR1; 70 nM), which binds actin filaments, both prepared in blocking solution. Incubations were performed for 1 h and 30 min, respectively, under gentle agitation and protected from light. Oocytes were then washed in PBS-T.

Oocytes (19 GV oocytes and 13 MII oocytes) were mounted on µ-Slide 18 Well Glass Bottom chambers using VECTASHIELD® PLUS Antifade Mounting Medium with DAPI (Vector Laboratories, H-2000). Confocal images were acquired on a Leica Stellaris 5 spectral confocal microscope (Leica Microsystems), and image analysis was performed using ImageJ2 v1.54p (NIH).

### RNA isolation and cDNA synthesis

Ovaries (16 in total) were obtained as described above (OVH, see above “*Collection and *In Vitro* Maturation of Cat Oocytes”).* Tissue was then sectioned, transferred into 1.5 mL Eppendorf tubes containing TRIzol reagent (Ambion), and stored at − 20 °C until processing.

RNA was extracted from ovarian tissue using the TRIzol–chloroform method. Ovaries were manually dissected, and 100 mg tissue fragments were transferred into microtubes containing 1 ml of TRIzol reagent (Ambion), and homogenized. Metaphase II (MII, 10 ovaries) eggs were obtained by culturing GV-stage oocytes in maturation (as previously described). Subsequently, RNA was isolated from oocyte samples, including germinal vesicle (GV, isolated from 12 ovaries) oocytes and MII eggs, using approximately 180 eggs per extraction with the ReliaPrep™ RNA Cell Miniprep System (Promega). Homogenates of the ovarian tissue were mixed with 200 µl of chloroform per 1 ml of TRIzol for 3 min at RT, and then centrifuged at 12,000 g for 10 min at 4 °C. The aqueous phase was transferred to a new tube, mixed with 500 µl isopropanol per 1 ml of TRIzol, incubated for 10 min at 4 °C, and centrifuged at 12,000 g for 10 min at 4 °C. The resulting RNA pellet was washed in 1 ml of 75% ethanol and centrifuged at 7,500 g for 5 min at 4 °C. After removing the supernatant, the pellet was air-dried for 5 min at 37 °C and resuspended in 50 µl nuclease-free water.

RNA concentration and purity were assessed using a Nano-400A spectrophotometer (ALLSHENG). cDNA synthesis was performed using the RevertAid First Strand cDNA Synthesis Kit (Thermo Fisher Scientific) according to the manufacturer’s instructions. cDNA products were stored at -20 °C until use.

### PCR for cTRPV3

Cat *TRPV3* cDNA sequences were retrieved from the Ensembl database (https://www.ensembl.org/). Primers were designed using the Primer-BLAST tool provided by the National Center for Biotechnology Information (NCBI, https://www.ncbi.nlm.nih.gov/tools/primer-blast/). The *SDHA* gene (Succinate dehydrogenase subunit A) was selected as the housekeeping gene for normalization [27]. Primer sequences are listed in Table 1.

**Table 1:** Primer sequences for *cTRPV3* and c*SDHA* genes

Gene	Primer	${\mathrm{Sequence}}\left( {5^{\prime} \to 3^{\prime}} \right)$	Tm (°C)	Length (bp)	Amplicon size (bp)
TRPV3	Forward	AGGAAGATTTCCGGCTGTGT	58	20	118
	Reverse	TTGTTGAAATCTGCTGTCCGT		21	
SDHA	Forward	GCTCTTTCCTACGAGATCACAC	58	22	112
	Reverse	GCCTTTCACAGTGTCGTAGAA		21	

Primer sequences used to amplify *TRPV3* and the reference gene succinate dehydrogenase complex, subunit A, *SDHA*, from *Felis catus* tissue and oocytes

PCR amplification was performed using synthesized cDNA as a template. Each reaction contained 10 × PCR buffer, 50 mM MgCl_2_, 10 mM dNTPs, 10 µM forward and reverse primers, 5 U/µl Taq DNA polymerase (Thermo Fisher Scientific), and 1 µl cDNA. The PCR program consisted of an initial denaturation at 94 °C for 5 min, followed by 35 cycles of denaturation at 94 °C for 40 s, annealing at 58 °C for 40 s, and extension at 72 °C for 30 s. A final extension at 72 °C for 10 min.

PCR products were resolved on 3% agarose gel stained with Midori Green Advance (NIPPON Genetics EUROPE) and visualized using an ultraviolet transilluminator.

### Calcium imaging of cat MII eggs

Intracellular calcium concentration ([Ca^2+^]_i_) in ZP-free MII eggs was measured using Fura-Red-AM (Invitrogen, Waltham, MA, USA). Eggs were incubated in 5 µM Fura Red-AM supplemented with 0.01% Pluronic F127 in high-glucose DMEM for 30 min at RT. After incubation, eggs were washed in the handling medium (Medium 199 supplemented with 18 mM Hepes and 10% FBS), and. transferred to an extracellular solution containing (in mM): 140 NaCl, 4 KCl, 2 CaCl_2_, 1 MgCl_2_, 10 HEPES, and 10 glucose, adjusted to pH 7.4 with NaOH. Calcium imaging was performed using a Leica Stellaris 5 spectral confocal microscope, acquiring images every 3 s for a total of 9 min.

Fura Red-loaded eggs were excited at 405 nm and 488 nm, corresponding to the Ca^2+^-bound and Ca^2+^-unbound forms of the dye, respectively. The emission of Fura Red–AM in the Ca^2+^ bound state was measured at 610 nm for the Ca^2+^-bound state and 670 nm for the unbound state. Fluorescence intensities were quantified using LAS X software (v.4.3.0.24308), and Ca^2+^-responses were expressed as the ratio of 405/488 nm fluorescence.

To assess TRPV3 channel activity, 2-Aminoethyl diphenylborinate (2-APB, Sigma-Aldrich, D9754) was applied at final concentrations of 100 µM or 300 µM [28]. Eggs (6 eggs) imaged in the absence of 2-APB and in the extracellular solution were used as controls. All imaging experiments were conducted at RT.

### Cat egg activation using 2-APB

GV oocytes were isolated and matured in vitro for 24–26 h at 38.5 °C in a humidified atmosphere containing 5% CO_2_ as described above (“*Collection and *In Vitro* maturation of Cat oocytes*”)_._ MII eggs exhibiting a visible first polar body were selected and incubated in maturation media to which 0.1% PVA was added instead of 0.3% BSA, supplemented with 300 µM 2-APB for 1 h at 38.5 °C and 5% CO_2_ to induce oocyte activation (24 eggs). After treatment, eggs were transferred to synthetic oviduct fluid (SOF) medium [29] containing 107.6 mM NaCl, 7.16 mM KCl, 1.19 mM KH_2_PO_4_, 2.23 mM CaCl_2_*2H_2_O, 0.472 mM MgCl_2_*6H_2_O, 25.07 mM NaHCO_3_, 0.251 mM sodium lactate, and phenol red. The SOF medium was further supplemented with 0.4 mM sodium pyruvate, 0.271 mM L-glutamine, 0.3% BSA, 1X MEM essential amino acids, 1X MEM non-essential amino acids, 10 ng/ml EGF, 0.1 mg/ml citric acid, 0.5 mg/ml Myo-inositol, and 2% FBS. Eggs were cultured in SOF for up to 40 h following 2-APB exposure. Control eggs (31 eggs) were processed identically, except for the absence of 2-APB treatment. Statistical differences between conditions were assessed using a one-way repeated measures ANOVA with Geisser–Greenhouse correction, followed by Sidak’s multiple comparisons test.

### (2)-APB docking in the cat TRPV3 channel

The cat TRPV3 channel model and the computational docking of 2-APB to the cat TRPV3 (cTRPV3) channel were generated using Boltz-2 [30, 31]. The amino acid sequence of *Felis catus* TRPV3 (NCBI accession number: XP_019672883.1) was used as input to generate a structural model of the channel. To evaluate ligand-channel interactions, we used Visual Molecular Dynamics software v1.9.4 [32]. Boltz-2 software was also used to estimate the predicted EC_50_ for 2-APB binding to cTRPV3.

To estimate the volume and structural flexibility of the 2-APB binding sites in mouse, human, and cat TRPV3 channels, we used the FPocket software package [33]. For each species, binding-site volumes were calculated for individual subunits, and the average value was reported. For the mouse TRPV3 (mTRPV3; PDB ID: 6DVZ), we quantified the average pocket volume across three regions previously described as potential ligand-binding sites: the extracellular, transmembrane, and intracellular domains. For the human TRPV3 (hTRPV3; PDB ID: 8V6N), average pocket volumes were measured for the transmembrane and intracellular binding sites, since the 2-APB does not bind the extracellular binding site.

The molecular volume of 2-APB was determined using the Molecular Volume Calculator webserver (https://scfbio-iitd.res.in/Sanjeevini/Molecular-volume-calculator.php accessed on October 27th, 2025) following the procedure described by Jayaram et al., [34].

Three-dimensional structural visualization of mouse, human, and cat TRPV3 channels was performed using Visual Molecular Dynamics (VMD) v1.9.4 [32]. This software was used to inspect overall channel architecture, compare pore and binding-site regions among species, and generate molecular graphics for analysis and figure preparation.

#### TRPV3 comparative sequence analysis

A comparative sequence analysis of TRPV3 from mouse, human, and domestic cat was conducted to identify species-specific differences within functionally relevant domains. Protein sequences for *Mus musculus* (TRPV3_MOUSE), *Homo sapiens* (TRPV3_HUMAN), and *Felis catus* (XP_019672883.1) were retrieved from UniProt and NCBI. Multiple sequence alignment was performed using MAFFT under the *auto* strategy to generate a high-quality alignment. Pairwise sequence identity and similarity across full-length TRPV3 proteins were calculated using the EMBOSS Needle webserver (https://www.ebi.ac.uk/jdispatcher/psa/emboss_needle).

Residue numbering was standardized to the mouse TRPV3 reference sequence to facilitate cross-species comparison of structural domains. Functional regions—including the ankyrin repeat domain (ARD), S1-S4 temperature-sensing helices, S4-S5 linker, pore-forming region (S5-pore helix-selectivity filter-S6), and TRP helix—were annotated based on published TRPV3 structural models and cryo-EM structures [35, 37]. For each domain, residue-by-residue comparisons were performed to identify conservative and non-conservative substitutions across species, with classification based on differences in physicochemical properties. Putative metal-coordinating residues (Cys, His, Asp, Glu) were specifically flagged due to their relevance in Ca^2+^ and Zn^2+^ permeation [38].

Domain-specific differences were organized into comparative matrices and subsequently used to interpret potential impacts on channel gating, thermal sensitivity, ion selectivity, and ligand binding. Structural alignments and domain visualizations were generated using Genious Prime.

## Results

### Cat MII eggs are larger than mouse eggs

**Fig. 1:**
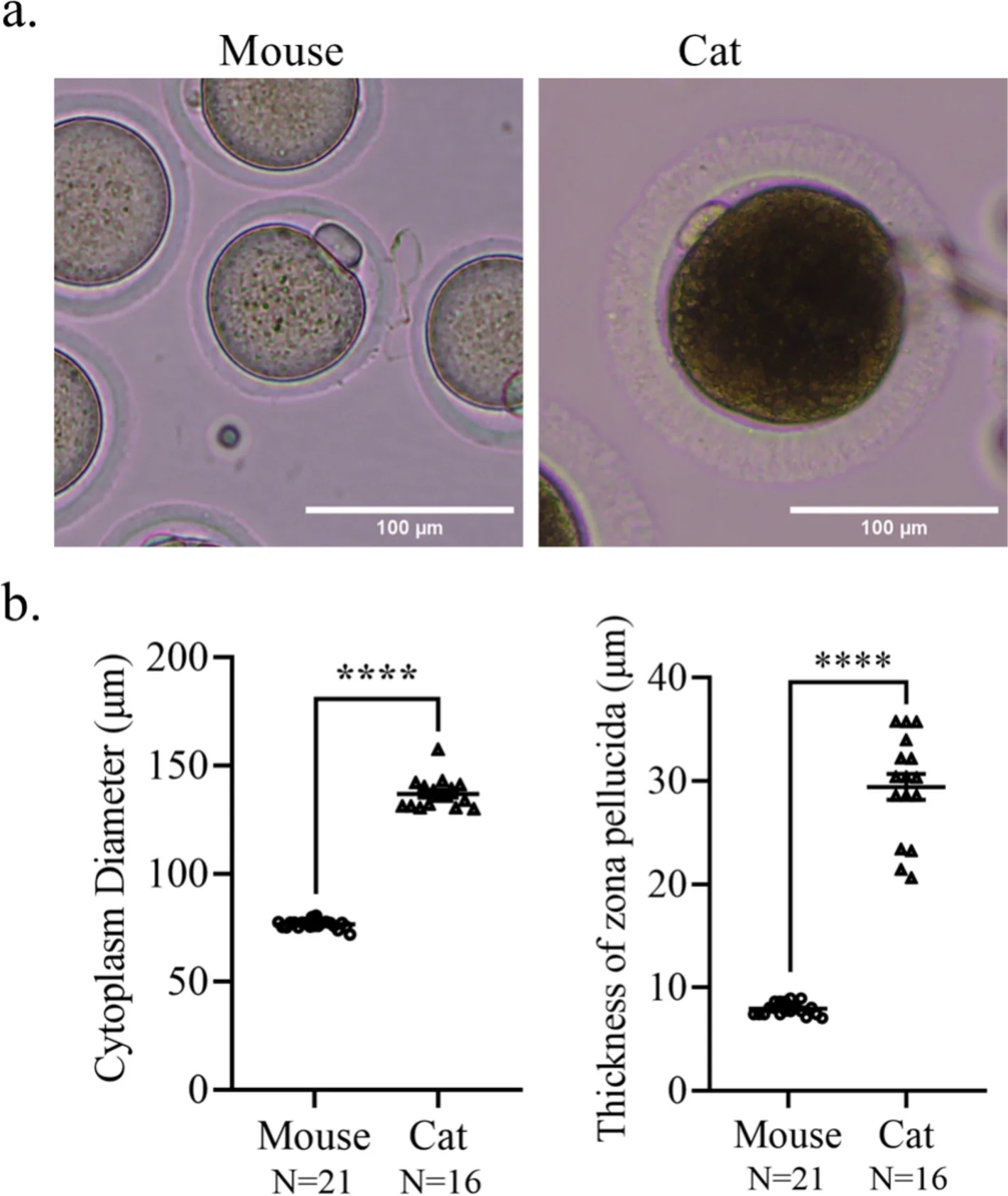
Cat oocytes are larger and show a thicker *zona pellucida* (ZP) than mouse oocytes. **a** Representative brightfield images of mature metaphase MII eggs from mouse (left) and domestic cat (right). **b** Quantitative comparisons show significant differences in cytoplasmic diameter (left panel) and ZP thickness (right panel) between species. The mean cytoplasmic diameter of mouse oocytes was 76.58 ± 0.4 μm (SEM), whereas cat oocytes measured 137 ± 1.7 μm. Differences were assessed using a Mann–Whitney test (*p* < 0.0001). ZP thickness was also significantly greater in cat oocytes (29.43 ± 1.25 μm) compared with mouse oocytes (7.94 ± 0.13 μm), as determined by an unpaired t-test with Welch’s correction (*p* = 0.0001). Sample sizes (N) are indicated in the figure

To assess the morphological characteristics of domestic cat oocytes, we measured the cytoplasmic diameter and zona pellucida (ZP) thickness of MII eggs and compared them with those of mice. Figure 1 presents a direct morphological comparison between mature metaphase MII eggs obtained from mouse and domestic cat, revealing marked interspecies differences in eggs size and *zona pellucida* (ZP) architecture representative brightfield images show that cat eggs displayed a darker, more opaque cytoplasm due to their lipid content, in contrast to the translucent appearance of mouse oocytes (Fig. 1a; [39]). Cat MII eggs had a significantly larger average cytoplasm diameter (137 ± 1.7 µm) compared to mouse MII eggs (76.58 ± 0.4 µm; Fig. 1b). In addition, the ZP of cat eggs was substantially thicker, averaging 29.43 ± 1.2 µm versus 7.9 ± 0.1 µm in mouse oocytes (Fig. 1b, right panel), corresponding to ~3,7-fold increase. Together, these data demonstrate clear species-specific differences in egg size and structural organization, underscoring the importance of considering morphological variability with developing or adapting assisted reproductive technologies across species.

### TRPV3 is expressed in cat ovaries, germinal vesicle (GV) oocytes, and metaphase II (MII) eggs

**Fig. 2:**
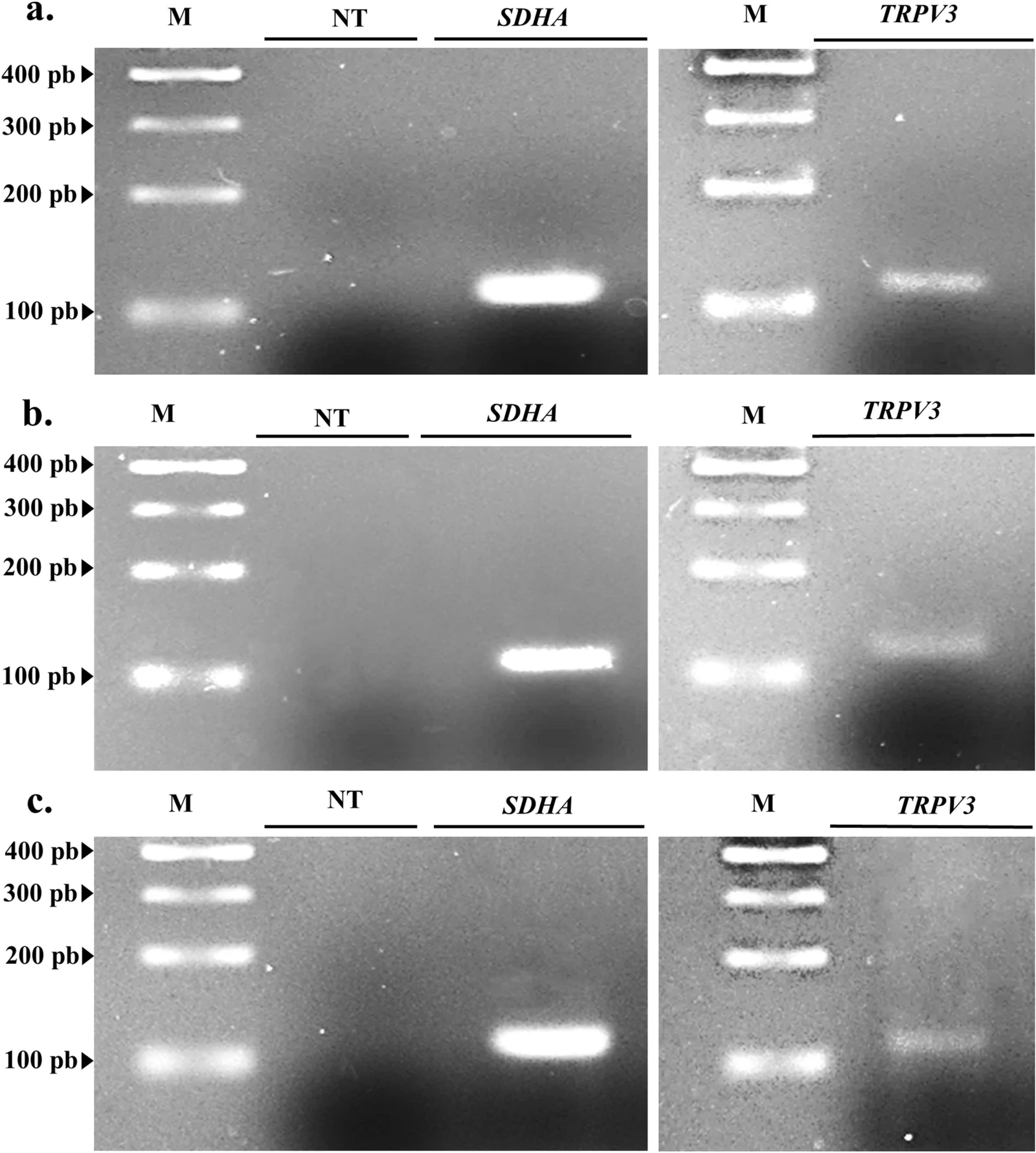
RT-PCR detection of *Trpv3* expression in domestic cat ovaries and oocytes at the GV and MII stages. Agarose gel electrophoresis of RT-PCR products obtained from cDNA derived from **a** domestic cat ovaries, **b** germinal vesicle (GV) oocytes, and **c** metaphase II (MII) eggs. M: 100 bp molecular weight marker. NT: no-template negative control. SDHA: housekeeping gene succinate dehydrogenase complex flavoprotein subunit A (112 bp). *Trpv3*: target gene (118 bp). Arrows indicate the expected amplicon sizes

Cation-permeable, non-selective ion channels play essential roles in oocyte maturation and fertilization, including TRPV3 [22, 26]. To determine whether *TRPV3* transcript is expressed in domestic cat reproductive tissues, we performed RT-PCR on RNA isolated from ovaries and oocytes/eggs at different developmental stages. In cat ovaries (Fig. 2a), amplification of the housekeeping gene *SDHA* yielded a band at the expected size of 112 pb. Under the same conditions, a distinct *TRPV3* amplicon of 118 pb was detected, indicating that *Trpv3* is transcriptionally expressed in domestic cat ovaries (Fig. 2a). Consistent with the ovarian data, *TRPV3* and *SDHA* transcripts were detected in both GV-stage oocytes and in vitro-matured MII eggs (Fig. 2b, c), demonstrating that *TRPV3* is transcribed throughout key stages of feline oocyte development.

### TRPV3 localizes to the plasma membrane of GV and MII feline oocytes

**Fig. 3:**
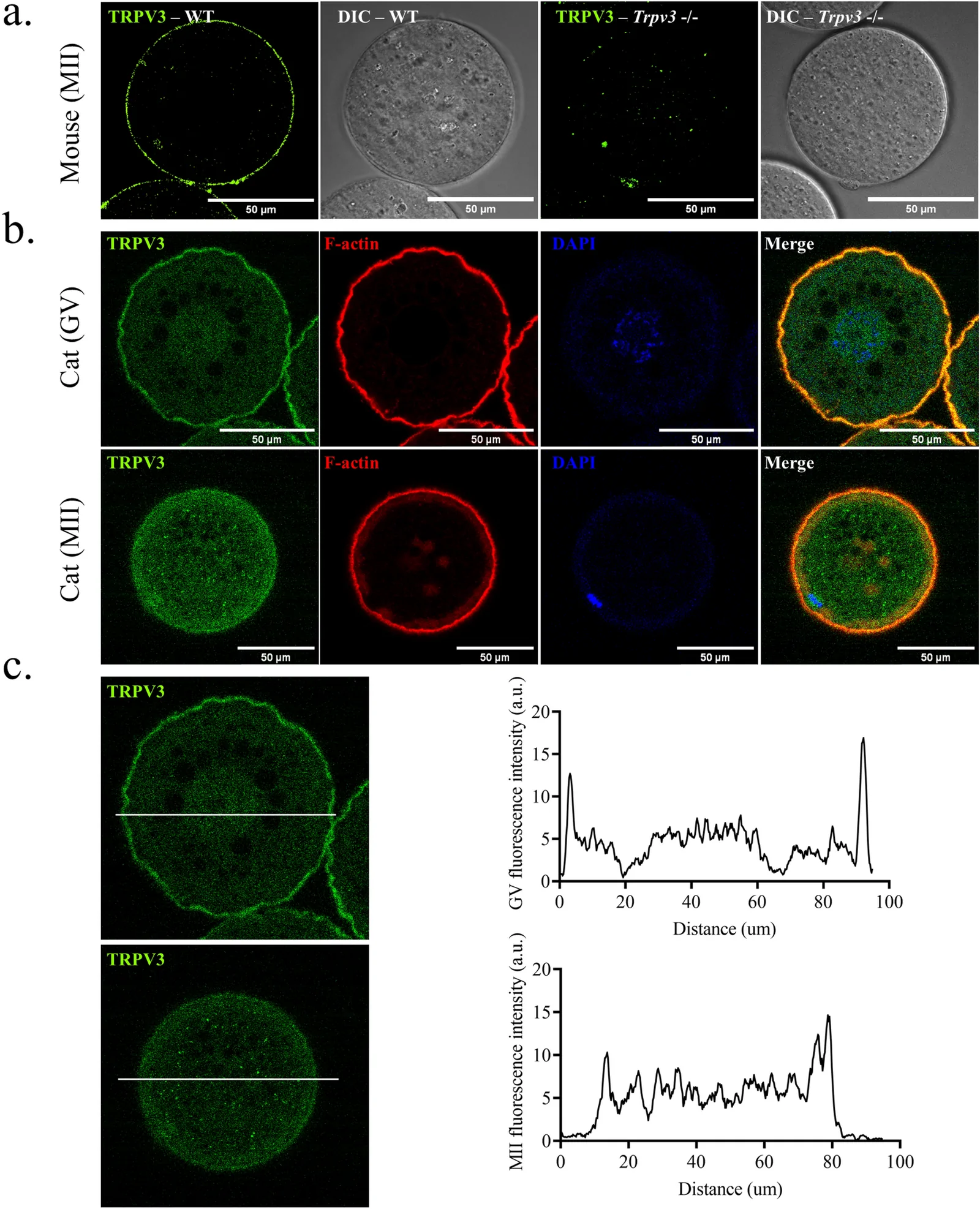
Plasma membrane localization of TRPV3 protein in feline oocytes and eggs. **a** Validation of anti-TRPV3 antibody specificity by immunostaining in C57BL/6 J mouse MII eggs from WT and TRPV3KO animals. WT oocytes (n = 24) displayed specific plasma membrane staining consistent with the TRPV3 localization. In contrast, TRPV3KO oocytes (n = 18) showed no detectable staining, confirming the absence of the protein and the specificity of the antibody. As a negative control, oocytes incubated with secondary antibody only (n = 10) exhibited no signal (data not shown) **b** Upper and lower panels: Representative images of feline oocytes at GV and MII stages stained with antibodies against TRPV3 (green), F-actin (red), and DNA (blue), along with merged channels. TRPV3 signal is observed in the cortical region of both GV (n = 19, upper panel) and MII (n = 13, lower panel) oocytes. DNA is localized centrally in GV oocytes and peripherally in MII oocytes, consistent with DNA arrangement. **c** Fluorescence intensity profiles across the oocyte diameter were generated to quantify TRPV3 signal distribution (white line in the images). In both GV (upper panels) and MII eggs (lower panels), TRPV3 fluorescence, measured as fluorescence intensity vs. distance (µm), is enriched at the plasma membrane

To determine whether the TRPV3 protein is expressed in feline oocytes, we performed immunofluorescence on GV- and MII-stage oocytes. To evaluate the specificity of the antibody, we examined TRPV3 expression in mouse MII eggs, as has been done previously [22]. In wild-type MII eggs, TRPV3 localized predominantly to the plasma membrane, whereas TRPV3 knockout (TRPV3^−/−^) eggs displayed no signal, confirming the specific recognition of the antibody, and providing support for its use in detecting TRPV3 in cat oocytes, although species-specific cross-reactivity cannot be completely excluded (Fig. 3a).

Next, TRPV3 localization was examined in domestic cat oocytes at different stages of meiotic maturation (Fig. 3b, c). In GV oocytes, TRPV3 immunoreactivity was concentrated in the plasma membrane and partially co-localized with cortical F-actin, in agreement with previous studies that showed that the functional expression of TRPV3 on the plasma membrane depends on an intact actin cytoskeleton [40]. Nuclear DNA staining (DAPI) was centrally localized as expected for GV-stage oocytes (Fig. 3b, upper panel). A weaker signal was detected within the cytoplasm (Fig. 3b, c). In MII-stage cat eggs, TRPV3 remained enriched in the plasma membrane, but cytoplasmic labeling was noticeably stronger than in GV oocytes (Fig. 3b, c). To quantitatively assess TRPV3 distribution, fluorescence intensity profiles were generated across the oocytes/eggs. In both GV oocytes and MII eggs, TRPV3 fluorescence peaked at the periphery and decreased toward the center (cell cytoplasm), indicating preferential localization at or near the plasma membrane (cortical area) (Fig. 3c, upper and lower right panels). Together, these results demonstrate that TRPV3 is a plasma membrane- associated channel in feline oocytes and that its localization undergoes dynamic redistribution during meiotic maturation.

### (2)-APB-sensitive Ca^2+^ influx in cat MII eggs is consistent with TRPV3 channel activity

**Fig. 4:**
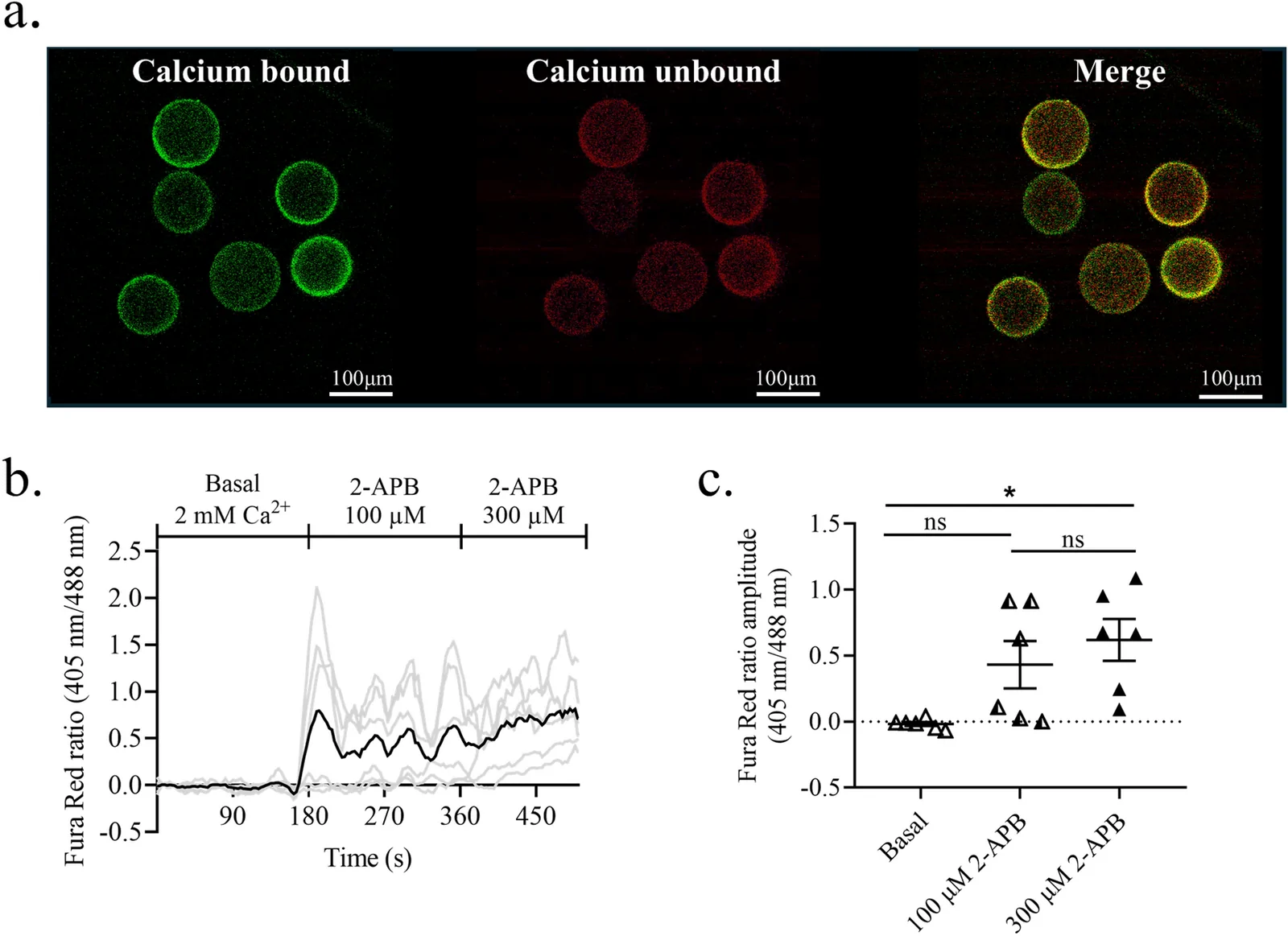
2-APB increases intracellular Ca^2+^ levels in cat MII eggs. **a** Representative confocal images of zona pellucida–free metaphase II (MII) cat eggs showing intracellular Ca^2+^ responses following application of 300 µM 2-APB, monitored using the ratiometric Ca^2+^ indicator Fura red. **b** Representative time courses of the Fura Red fluorescence ratio (405/488 nm) recorded from individual MII eggs under basal conditions and after application of 100 µM 2-APB at 3 min and 300 µM 2-APB at 6 min. Individual responses are shown in gray, and the mean trace is shown in black (n = 6 eggs). **c** Quantification of the mean amplitude of the Fura Red fluorescence ratio under basal conditions and following application of 100 µM or 300 µM 2-APB. Data are presented as mean ± SEM from six independent experiments (n = 6). Statistical differences between conditions were assessed using a one-way repeated measures ANOVA with Geisser–Greenhouse correction, followed by Sidak’s multiple comparisons test. A significant increase in intracellular Ca^2+^ levels was observed in response to 300 µM 2-APB compared with basal conditions (*p = 0.0224), whereas no significant differences were detected between basal and 100 µM 2-APB or between 100 µM and 300 µM 2-APB

To evaluate the functional activity of TRPV3 channels in domestic cat oocytes, we performed calcium imaging on ZP-free MII eggs loaded with the ratiometric calcium indicator Fura Red-AM (Fig. 4). Representative confocal images illustrate changes in intracellular Ca^2+^ levels following exposure to 300 μM 2-APB (Fig. 4a). Under these conditions, eggs displayed a clear shift in Fura red fluorescence consistent with an increase in cytosolic Ca^2+^, as indicated by enhanced calcium-bound signal and corresponding changes in merged images (Fig. 4a).

Under control conditions, in the absence of agonist, oocytes showed no variations in fluorescence values, indicating a stable basal Ca^2+^ (Fig. 4b, c). In contrast, stimulation with 2-aminoethoxydiphenyl borate (2-APB), a well-characterized TRPV3 agonist [28], induced dose-dependent robust Ca^2+^ increases. Exposure to 100 µM 2-APB produced a change in fluorescence (ΔF = 0.4319 ± 0.1789; Fig. 4b, c), which was improved upon by 300 µM 2-APB (ΔF = 0.6198 ± 0.1585; Fig. 4b, c). Individual eggs are shown in gray, while averaged response highlights the overall trend across the experiments (Fig. 4b). The mean Fura red fluorescence ratio under basal conditions did not differ significantly from that recorded following application of 100 μM 2-APB. In contrast, exposure to 300 μM 2-APB resulted in statistically significant increase in intracellular Ca^2+^ levels compared to basal conditions (Fig. 4c).

These results suggest that TRPV3 channels in cat MII eggs are functionally active and responding to pharmacological activation with a stereotypical elevation in intracellular Ca^2+^.

### (2)-APB-induced parthenogenesis in cat MII eggs is consistent with TRPV3 channel activation

**Fig. 5:**
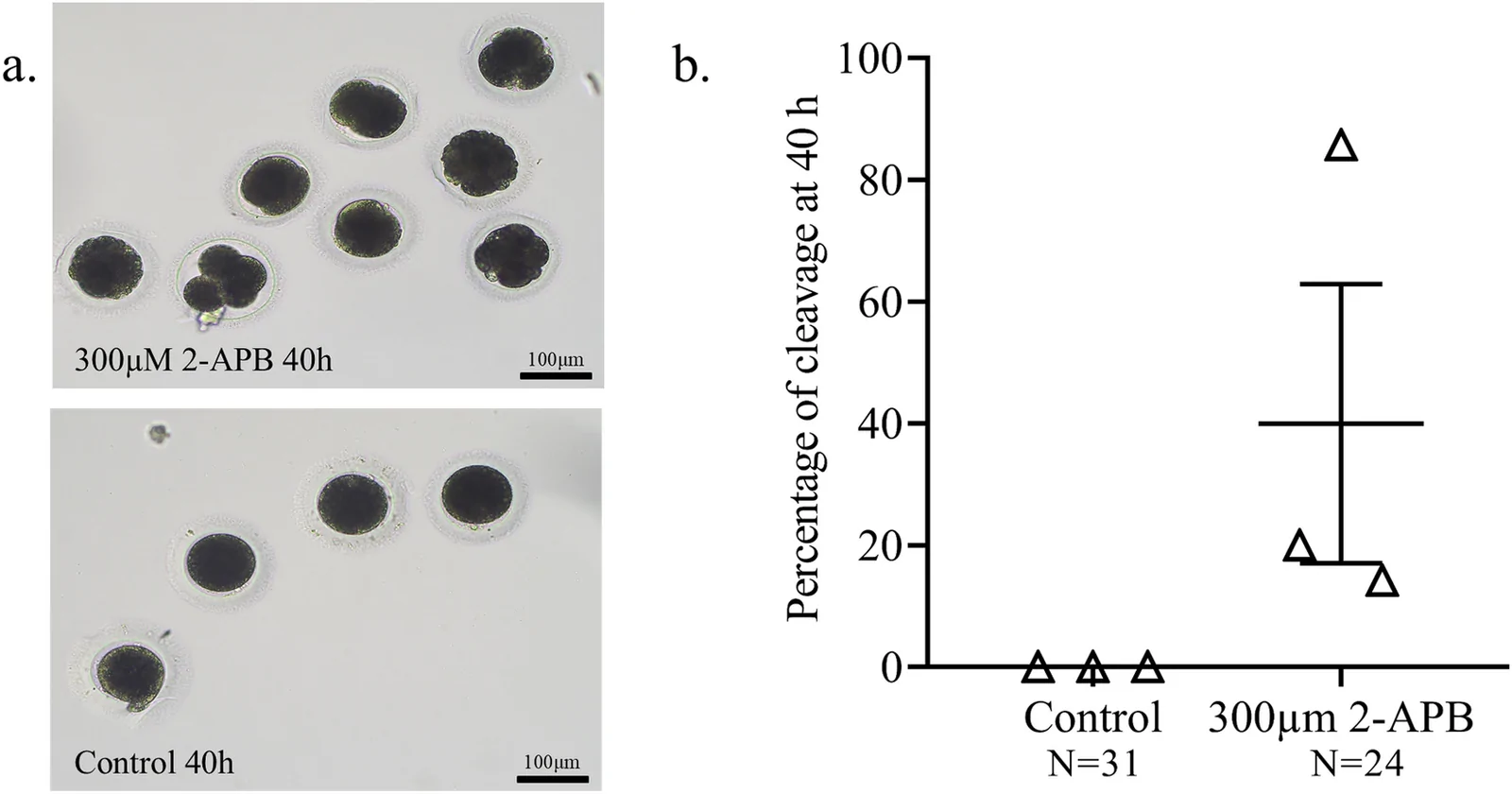
2-APB induces activation of cat MII eggs. **a** Representative brightfield images of control eggs (bottom) and eggs treated with 300 M 2-APB (top) acquired 40 h after agonist incubation. **b** Percentage of cleaved embryos 40 h after activation with 300 µM 2-APB for 1 h at 38.5 °C. Data represents three biological replicates; the number of oocytes (n) is indicated in the graph. Values are shown as mean ± SEM

We next investigated whether 2-APB induced Ca^2+^ influx is sufficient to promote parthenogenetic activation in domestic cat MII eggs as it does in mouse eggs mediated by TRPV3 channel activation [22]. We examine whether TRPV3 pharmacological stimulation with 2-APB is sufficient to induce egg activation and early cleavage in domestic cat MII eggs. Egg activation was assessed morphologically, quantifying cleavage rates following the agonist treatment. Representative brightfield microscopy images acquired 40 h after 2-APB treatment illustrate differences between control and eggs exposed to 2-APB (Fig. 5a). In the absence of 2-APB, control oocytes showed no signs of activation in any of the replicates, confirming that spontaneous activation did not occur under our experimental conditions. In contrast, incubation of MII eggs with 300 µM 2-APB resulted in a measurable activation response evidenced as cleavage. Eggs exposed to 2-APB for 60 min showed a low but consistent activation measured as % of cleavage at 40 h (39,97 ± 22,93%; Fig. 5a, b). These results suggest that activation of TRPV3 channels by 2-APB generates sufficient Ca^2+^ influx to trigger parthenogenetic activation in cat eggs. However, in contrast to mouse MII eggs [22], activation of cat MII oocytes by 2-APB required an extended incubation and higher agonist concentrations, highlighting species-specific differences between cat and mouse in TRPV3 function or the mechanisms of MII arrest.

### Comparative structural analysis of TRPV3 orthologs reveals conserved and divergent 2-APB binding pockets.

**Fig. 6:**
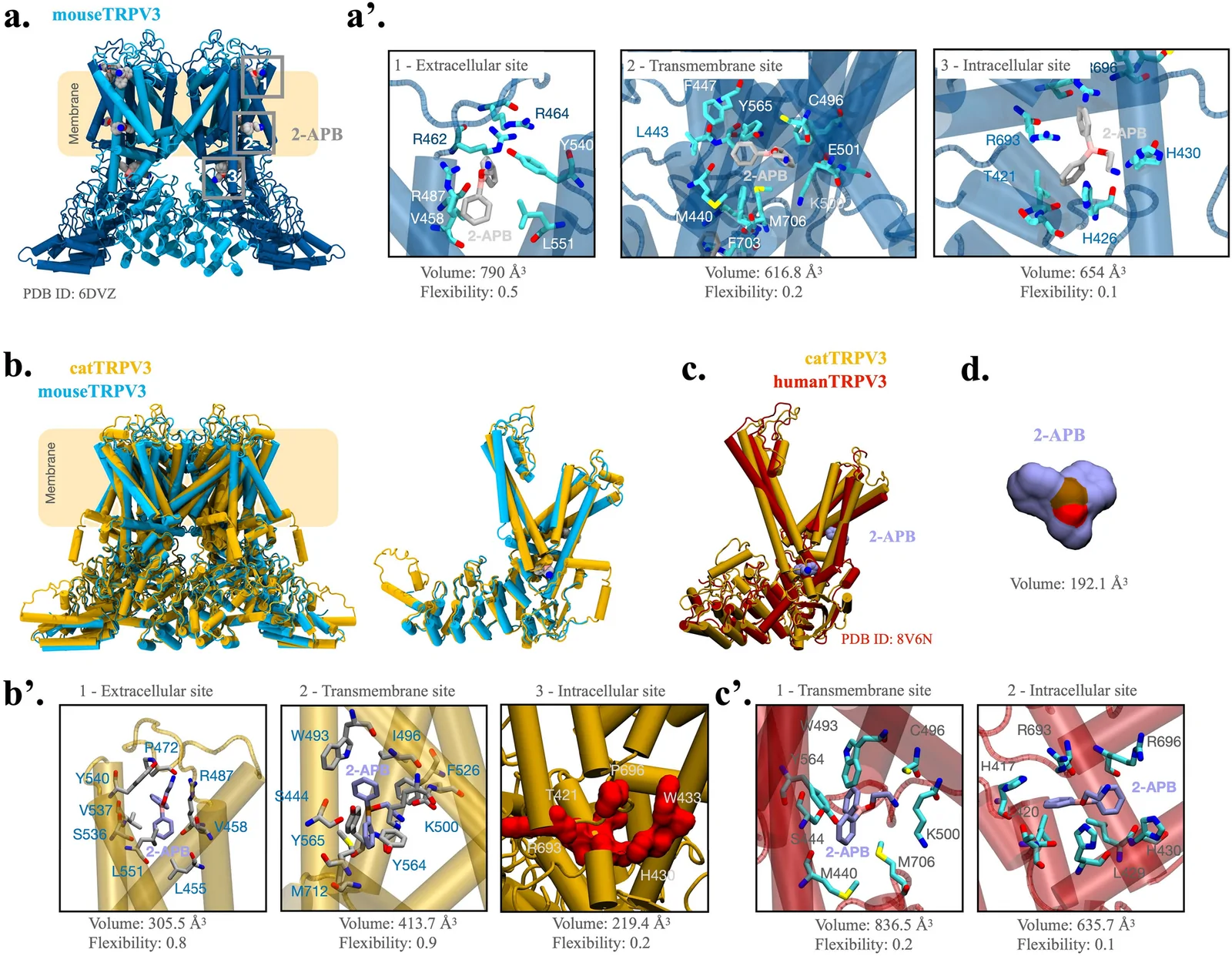
Comparative analysis of TRPV3 orthologs and predicted 2-APB binding sites. **a** Structural representation of the mouse TRPV3 channel (PDB ID: 6DVZ) docked with 2-APB. Three binding sites per subunit—extracellular, transmembrane—were identified. **a’** Close-up views of each binding pocket, showing residues predicted to interact with 2-APB. Calculated pocket volumes and flexibilities are indicated below each panel. **b** Structural superposition of cat TRPV3 (yellow) and mouse TRPV3 (cyan). Left: overall channel architecture. Right: single-subunit overlay illustrating strong conservation of the transmembrane region. **b’** Predicted 2-APB binding pockets in cat TRPV3 based on molecular docking. The extracellular and transmembrane cavities are accessible, whereas the intracellular cavity is substantially smaller than in mouse TRPV3, indicating steric limitations for 2-APB binding. **c** Structural comparison of cat TRPV3 (yellow) and human TRPV3 (red; PDB ID: 8V6N), showing conserved global architecture and ligand-binding regions. **c’**. Close-up views of the human TRPV3 binding pockets, highlighting residues previously implicated in 2-APB interactions, along with calculated volumes and flexibilities. **d** Molecular surface representation and calculated volume of the 2-APB molecule

To explore structural bases for species features in 2-APB sensitivity, we compared mouse, cat, and human TRPV3 by structural molecular modeling and docking, using the mouse cryo-EM structure (PDB 6DVZ) as a reference (Fig. 6a, [41]). Mouse TRPV3 contains three 2-APB pockets per subunit, located in the extracellular, transmembrane pocket, and intracellular, with distinct volume and conformational flexibilities; the transmembrane site is the most geometrically restricted (Fig. 6a’). The predicted cat TRPV3 (yellow) largely preserves the overall mouse channel architecture (Fig. 6b, cyan), but shows marked differences in pocket geometry. Notably, the cat intracellular pocket is considerably smaller (219.4 Å^3^, Fig. 6b’ right) than the mouse intracellular pocket (654 Å^3^, Fig. 6a’ right), rendering it unlikely to accommodate 2-APB (Fig. 6b’).

Comparison between cat and human TRPV3 (PDB ID: 8V6N) structures (Fig. 6c) further supported the conservation of global architecture and key ligand-interacting region. A close-up analysis of the predicted human TRPV3 2-APB binding pockets showed that residues previously involved in 2-APB interactions are largely conserved (Fig. 6c’). Given the estimated molecular volume of 2-APB (~ 192.1 Å^3^; Fig. 6d), the structural model predicts that only the extracellular and transmembrane sites in cat TRPV3 are predicted to be sterically permissive. Boltz‑2 EC50 predictions support this structural interpretation: mouse extracellular, transmembrane, and intracellular pockets gave EC50s of 0.26, 3.5, and 11.02 µM, respectively, whereas cat TRPV3 shows EC50s of 0.52 µM (extracellular) and 3.4 µM (transmembrane) and no accessible intracellular site; predicted human values are 2.45 µM (transmembrane) and 7.6 µM (intracellular) (Table 2).

**Table 2:** Predicted EC50 values for 2-APB at the mouse (mTRPV3), cat (cTRPV3), and human TRPV3 (hTRPV3)

	EC50 predicted (µM)		
	mTRPV3	cTRPV3	hTRPV3
Extracellular site	0.26	0.52	–
Transmembrane site	3.5	3.4	2.45
Intracellular site	11.02	–	7.6

### Comparative sequence and structural analysis reveals species-specific determinants in TRPV3 architecture and temperature-sensing domains

To identify additional relevant molecular determinants that could account for the differential 2-APB responses of cat, mouse, and human TRPV3 channels, we performed a comparative sequence and structural analysis sequence analysis focusing in the pore-forming and in the temperature-sensing/gating modules.

**Fig. 7:**
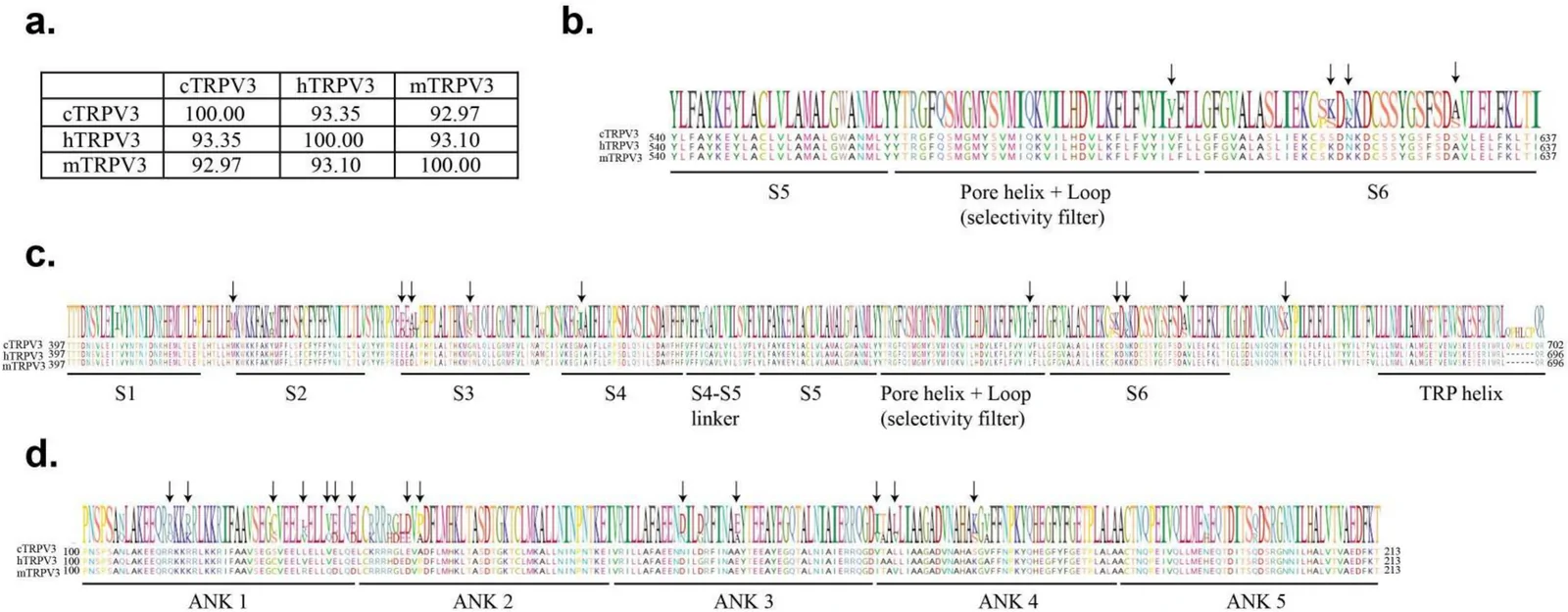
Comparative sequence analysis of mammalian TRPV3 orthologs reveals species-specific amino acid substitutions in the pore and temperature-sensing domains. **a** Pairwise sequence identity among mouse, human, and domestic cat TRPV3 channels, reveals high overall sequence conservation. **b** Multiple sequence alignment of the pore-lining S6 helix, highlighting three non-conservative substitutions uniquely present in the cat TRPV3 ortholog. **c** Mapping of non-conservative substitutions within the ankyrin repeat domain (ARD), a region involved in thermal priming and allosteric modulation of the channel function. **d** Multiple sequence alignment of the temperature sensing S1-S4 transmembrane bundle, the S4-S5 linker, and the TRP helix, reveals further amino acids differences between the mouse and cat orthologs, indicated by arrows. These regions are critical for mediated activation

Overall pairwise sequence identity revealed a high degree of similarity across all species, reaching 93.10% between mouse-human, 92.97% between mouse-cat, and 93.35% between human-cat TRPV3 (Fig. 7a), indicating strong evolutionary conservation among mammalian TRPV3 orthologs. However, this string also indicates that species-specific differences in channel behavior are likely driven by a limited number of strategically positioned substitution rather than by a large-scale structural divergence.

Detailed inspection of the pore forming S6 transmembrane helix revealed four non-conservative amino acid substitutions in the cat TRPV3 channel relative to the mouse counterpart. The changes, L596V, K614S, K616N, and A628S (Fig. 7b; Supplementary Table 1) localize to the lower S6 gate, a critical region that couples ligand binding to the pore opening [35]. In mouse TRPV3, the adjacent lysines K614-K616 form a localized positive patch that likely stabilizes the open state and facilitates allosteric transitions induced by 2-APB. In contrast, the substitution of these residues by neutral amino acids S614 and N616 in cat TRPV3 sequence eliminated this electrostatic feature, likely altering local charge distribution, hydration, and conformational flexibility of the activation gate. In addition, the A628S substitution introduces increased polarity near the pore, which may further influence S6 stability and gating dynamics.

Beyond the pore region, multiple non-conservative substitutions were identified between mouse and cat TRPV3 within the domains involved in temperature-sensing and allosteric regulation, including the ankyrin repeat domain (ARD), the S1-S4 bundle, S4-S5 linker, and the TRP helix. Notable differences include Q114R, C131S, R136L, D198N, E207A, K246S, D468A, and S480G (Supplementary Table 2; Fig. 7). Substitutions within the ARD (Fig. 7d) may affect thermal priming, while changes within the S1-S4 bundle and S4-S5 linker (Fig. 7c) directly influence the gating apparatus that 2-APB stabilizes in mouse TRPV3. Together, the clustered substitutions in the S6 gate and in temperature-sensing modules of cat TRPV3 provide a plausible molecular basis for its reduced sensitivity to 2-APB compared with mouse and human orthologs.

## Discussion

In this study, we demonstrate for the first time, that domestic cat oocytes express functional TRPV3 channel at both the mRNA and protein levels. Moreover, stimulation with the TRPV3 agonist 2-APB evokes robust Ca^2+^ influx in MII eggs, suggesting that TRPV3 protein is not only present but also functionally active at this developmental stage. This finding extends our previous work from mice [22, 26, 40] to a felid species.

In agreement with previous studies [1, 42], our quantitative analysis showed that cat MII eggs had a significantly larger average cytoplasm diameter (137 ± 1.7 µm) compared to mouse MII eggs (76.58 ± 0.4 µm; Fig. 1b). This ~ 1.8-fold increase in egg size was statistically significant, indicating a clear separation between the two populations. Feline MII eggs displayed larger diameters and thicker *zona pellucida* compared with mouse oocytes. These morphological differences likely reflect species-specific adaptations in feline reproductive physiology. The high lipid content characteristic of cat oocytes may contribute to energy storage and early embryonic development, both of which are associated with embryo competence and cryotolerance [43, 45]. A larger cytoplasmic volume may also imply greater Ca^2+^-buffering capacity and distinct organelle organization [46]. Such features could influence the amplitude and kinetics of Ca^2+^ signals during maturation and activation, helping explain why activation protocols developed for other species often perform sub optimally in felines. Consistent with this, conventional oocyte artificial activation treatments that are highly effective in mouse eggs have yielded only limited success in cat oocytes [19].These distinctive morphological features likely reflect species-specific reproductive adaptations in felids.

Calcium-permeable ion channels play essential roles in oocyte maturation and fertilization [12, 22, 40]. Our results on TRPV3 protein localization dynamics during cat oocyte maturation are similar with previous findings in mice, in which TRPV3 functional expression peaks at the MII stage [22]. TRPV3^−/−^ mice exhibit somewhat reduced ER Ca^2+^ stores and lower levels of labile Zn^2+^ [26], underscoring the importance of this channel for divalent cation homeostasis and fertilization [47]. In cat oocytes, TRPV3 is localized to both the cytoplasm and plasma membrane in MII eggs, however, and different from mouse oocytes, this pattern becomes more prominently membrane-associated in GV oocytes (Fig. 3). Moreover, cat GV oocytes displayed a more sharply defined cortical enrichment compared to MII eggs, different that the TRPV3 functional pattern observed in mouse GV oocytes [22]. This pattern, and its colocalization with actin, suggests dynamic trafficking of TRPV3 during maturation, as it was reported in mouse [40], and supports a functional role at fertilization. It is important to note that the antibody used in this study has not been validated in cat; therefore, the possibility of non-specific binding cannot be excluded. This limitation may provide plausible explanation for the observed discrepancies in membrane-associated TRPV3 localization between mouse and cat GV oocytes.

We demonstrate that the TRPV3-agonist 2-APB induces physiologically relevant Ca^2+^ elevations sufficient to trigger parthenogenetic activation in a subset of cat oocytes. However, activation rates were modest compared with those reported in other models [22] (Figs. 4, 5). This discrepancy can be explained by two key factors. First, successful egg activation in mammals typically requires not a single sustained Ca^2+^ rise but a series of repetitive Ca^2+^ oscillations [48]. A limitation of using 2-APB is that it primarily induces a non-oscillatory Ca^2+^ increase, TRPV3 activation alone may be insufficient to support full embryonic activation in all oocytes. In addition, the total Ca^2+^ rise induced by 2-APB requires the contributions of intracellular channels, such as the IP_3_R1, to propagate the Ca^2+^ wave initiated by the Ca^2+^ influx. However, the sensitivity of this channel in cats is presently unknown. Similarly, the permeability ratios of feline TRPV3 remain unknown, particularly for Sr^2+^_,_ whose influx through TRPV3 is known to trigger parthenogenetic activation in mouse eggs [22]. Second, our results suggest that species-specific structural differences in the TRPV3 protein may alter channel sensitivity to 2-APB [41, 49, 50]. Minor divergences in channel structure can substantially influence agonist responses. Thus, the comparatively limited response of feline vs. mouse eggs to 2-APB likely reflects an inherent pharmacological difference between species, on top of the suboptimal Ca^2+^ signal pattern. This interpretation agrees with prior reports that small structural changes can substantially alter TRPV3 sensitivity and efficacy to 2-APB [50]. These findings underscore the need to optimize agonist dose and exposure for each species and to seek TRPV3 modulators better matched to felid channels.

Our *in-silico* structural and docking analyses provided molecular basis for the reduced 2-APB sensitivity of cat TRPV3. As it was reported, our structural prediction of mouse TRPV3 contains three potential ligand-binding pockets: extracellular, transmembrane, and intracellular [41], whereas the cat TRPV3 structural model features a markedly smaller intracellular pocket relative to mouse TRPV3, and it is predicted to be inaccessible to 2-APB (Fig. 6). Consequently, the structural model predicts that cat TRPV3 has only two accessible 2-APB-binding pockets. Together these analyses suggest that while the transmembrane binding pocket is broadly conserved across species, and that species-specific structural differences, particularly within the intracellular cavity, may influence the accessibility and contribution of individual binding sites to 2-APB-mediated modulation of TRPV3. Indeed, cat TRPV3 likely has only two functional 2‑APB binding pockets per subunit (extracellular and transmembrane), and that modest changes in pocket volume and shape across orthologs can produce measurable differences in ligand sensitivity. This reduction in the binding-sites number or accessibility could diminish cooperative channel activation, effectively lowering the apparent agonist efficacy. Such replacements alter local charge distribution, hydration, and flexibility of the activation gate, offering a structural explanation for the reduced 2-APB sensitivity observed in cat oocytes. Together, these S6-specific substitutions support the notion that the 2-APB-stabilized conformation in mouse TRPV3 is not fully preserved in the cat ortholog, helping to explain differences in activation strength. In practical terms, feline TRPV3 might require higher 2-APB concentrations (or longer exposure) to achieve the same degree of channel opening and Ca^2+^ influx observed in the mouse with lower doses. Our results show that when designing artificial oocyte activation treatments for felids, simple adopting concentrations effective in mouse may be insufficient; instead, higher agonist doses or alternative TRPV3 modulators based in the particular structural characteristics of cat TRPV3, might be required. These findings underscore the need to optimize agonist dose and exposure for each species and to seek TRPV3 modulators better matched to felid channels.

Beyond ligand-binding sites, comparative sequence analysis of the domestic cat TRPV3 channel revealed several non-conservative amino acid substitutions that map to critical regions involved in channel gating and temperature sensing (Fig. 7). Several of these changes modify charge, polarity, or structural rigidity at positions previously implicated in heat sensitivity and allosteric coupling [36, 37], and could therefore modulate the energetics of temperature-dependent activation. Notably, four changes in the S6 helix of feline TRPV3 alter the electrostatic landscape of the lower pore domain. This region is essential for channel opening and for coupling ligand binding to pore dilation during activation [37]. Changes here could affect how the channel pore transitions from close to open states. We also identify unique substitutions across the ankyrin repeat domain, the S1-S4 transmembrane bundle (which acts as the temperature-sensing module), and the S4-S5 linker that connects the voltage-sensing like domain to the pore opening [51, 52]. The S4-S5 linker is often linkened to a mechanical “gearbox” in TRP channels translating conformational rearrangements in the sensor domain to the opening of the pore [53, 54], and upon activation by small-molecule agonists such as 2-APB [28, 50]. Therefore, the D468A substitution disrupts key electrostatic interactions in the linker region, which are required for efficient mechanical coupling between temperature-sensing helices and the S6 gate. Alterations of these domains in the cat TRPV3 likely engender subtle shifts in the energetics and kinetics and the stability of open or sensitized channel states. It is known from structural and biophysical studies that the heat activation of TRPV3 channel involve concerted conformational rearrangements in the S1-S6 transmembrane region [37, 52]. For example, TRPV1 (a heat-gated cousin of TRPV3) undergoes specific pore conformational changes upon heating and capsaicin binding [53, 54]. This molecular scenario provides a rational molecular explanation for the attenuated responsiveness to 2-APB observed in our Ca^2+^ imaging experiments. Collectively, these findings show that although TRPV3 is highly conserved across mammals, subtle yet strategically positioned sequence differences can significantly alter channel behavior in felids. This insight is particularly relevant for the development of TRPV3-based artificial oocyte activation strategies, emphasizing the need to consider species-specific pharmacodynamics when designing protocols for both domestic and endangered cats.

Collectively, these differences are predicted to alter thermal activation threshold, shift gating energetics, and generate species-specific conformational responses upon ligand binding.across species. Moreover, the substitutions we observe could bias permeation toward other divalent cations in addition to Ca^2+^, highlighting a potential role for ion-selectivity shifts in adaptive changes to mammalian physiology.

The broader implications of our work pertain to improving felid ARTs and acknowledging the current limitations in our understanding of felid egg activation. The efficiency of interspecific somatic cell nuclear transfer (iSCNT) in felids remains low, partly due to the limited understanding of domestic cat physiology and activation mechanisms [8]. Our study addresses one aspect of this knowledge gap by elucidating a potential Ca^2+^ signaling mechanism in cat oocytes. However, it also reveals limitations that need to be overcome. For instance, while triggering Ca^2+^ release via TRPV3 is a promising strategy (given TRPV3´s role during artificial egg activation, as shown here and in mice), it may need to be combined with techniques to mimic physiological Ca^2+^ oscillations [48] or supplemented by targeting other Ca^2+^ influx pathways to achieve full activation and embryonic development. Furthermore, oocytes from wild felids can differ in morphology and development responses, as demonstrated in the kodkod (*Leopardus guigna*), which produced smaller oocytes and exhibits species-specific sensitivities in activation protocols [10]. Such differences caution against directly extrapolating our results to all felids. A deeper understanding of Ca^2+^ signaling pathways, particularly the role of TRPV3 and how it structure varies between the species, will be essential for developing more effective and tailored activation protocols. Future work should build on this by exploring other Ca^2+^-permeable channels (e.g. TRPM7, Cav3.2) [55] that also are involved in egg activation, and by testing alternative TRPV3 agonist or modulators that might induce more favorable intracellular Ca^2+^ increases in felids eggs.

Our findings contribute to increasing the success rate of felid embryo production by identifying TRPV3 as viable target for artificial oocyte activation in felids and by illuminating the species-specific nuances that must be accounted for. By integrating molecular insights with reproductive technology, we move closer to establishing reliable assisted reproductive protocols not only for domestic cats but also as a model to safeguard the genetic future of endangered felines. This is particularly relevant for species such as the *kodkod*, for which gamete collection from live individuals is difficult and poses significant risks due to their small population size and protected status.

## Conclusions

In this study, using a combination of mRNA expression, functional assays and comparative structural analysis, we found that the cat TRPV3 ortholog appears to contain only two accessible ligand-binding pockets per subunit (compared to three in mouse channels) and harbors unique amino acid substitutions in key pore-forming and regulatory domains. These structural differences correlate with a markedly reduced 2-APB responsiveness in the cat channel, providing a mechanistic explanation for its diminished activation relative to the highly conserved mouse TRPV3. Our findings demonstrated that subtle sequence variations can substantially alter ion channel gating and ligand sensitivity across species, representing the principal contribution of this work.

Together, these findings provide the first evidence for TRPV3 in felid oocytes and eggs, and lay the groundwork for developing more effective, field-specific oocyte activation strategies in the context of ART and biodiversity conservation.

## Supplementary Information

Supplementary Material 1.

Supplementary Material 2.

## Data Availability

All data generated or analysed during this study are included in this published article and its supplementary information files.

## Abbreviations

2-APB: 2-Aminoethyl diphenylborinate
ARD: Ankyrin repeat domain
ARTs: Assisted reproductive technologies
cTRPV3: Cat TRPV3
CGs: Cortical granules
EGF: Epidermal growth factor
E₂: Estradiol
GV: Germinal-vesicle
TL-HEPES: HEPES-buffered tyrode lactate
hCG: Human chorionic gonadotropin
hTRPV3: Human TRPV3
IVC: In vitro culture
IVP: In vitro embryo production
IVF: In vitro fertilization
IVM: In vitro maturation
IUCN: International union for conservation of nature
iSCNT: Interspecific somatic cell nuclear transfer
[Ca^2^^+^]i: Intracellular calcium concentration
ICSI: Intracytoplasmic sperm injection
MII/eggs: Metaphase II
mTRPV3: Mouse TRPV3
OVH: Ovariohysterectomy
PMSG: Pregnant mare serum gonadotropin
SDHA: Succinate dehydrogenase subunit A) gene
SCNT: Somatic cell nuclear transfer
SOF: Synthetic oviduct fluid
TRPV3^-/-^: TRPV3 knockout, TRPV3KO.
VMD: Visual molecular dynamics
ZP: Zona pellucida
