# Supplementary material for: Expression and functional characterization of the TRPV3 channel in domestic cat (Felis catus) mature oocytes
Source: Biol Res. 2026 Jul 6;59:53. doi: 10.1186/s40659-026-00709-7 (PMC13621635; doi:10.1186/s40659-026-00709-7)
Supplement: Supplementary file 1 [file 40659_2026_709_MOESM1_ESM.pdf]

Supplementary Table 1. Non-conservative amino acid substitutions in the pore region of the TRPV3 ortholog from mouse, human, and domestic cat

Mouse position	Mouse (aa)	Human (aa)	Cat (aa)	Substitution type (human vs mouse)	Substitution type (cat vs mouse)	Domain	Note
596	L	V	V	Conservative	Conservative	Selectivity filter	Conservative hydrophobic substitution; likely preserves helix packing and pore gating properties
613	S	P	S	Non-conservative	Identical	S6	Non-conservative substitution in human may affect pore size or ion selectivity
614	K	K	S	Identical	Non-conservative	S6	Substitution from K to S in cat may alter charge properties of the pore, affecting gating
616	K	N	N	Non-conservative	Non-conservative	S6	Both human and cat have non-conservative substitutions at this position, likely altering gating
628	A	A	S	Identical	Non-conservative	S6	Non-conservative change in cat could affect selectivity and ion permeability
