# Supplementary material for: Expression and functional characterization of the TRPV3 channel in domestic cat (Felis catus) mature oocytes
Source: Biol Res. 2026 Jul 6;59:53. doi: 10.1186/s40659-026-00709-7 (PMC13621635; doi:10.1186/s40659-026-00709-7)
Supplement: Supplementary file 2 [file 40659_2026_709_MOESM2_ESM.pdf]

Supplementary Table 2. Non-conservative amino acid substitutions in temperature-sensing regions of the TRPV3 ortholog from mouse, human, and domestic cat

Mouse position	Mouse (aa)	Human (aa)	Cat (aa)	Substitution type (human vs mouse)	Substitution type (cat vs mouse)	Domain	Note
106	N	Q	N	Conservative	Identical	ANK 1	Conservative substitution in human; unlikely to strongly affect channel function
114	Q	R	R	Non-conservative	Non-conservative	ANK 1	Charge gain in both human and cat; may affect regulatory interactions in the N-terminus
117	K	R	R	Conservative	Conservative	ANK 1	Conservative basic substitution; likely preserves electrostatic properties
131	C	C	S	Identical	Non-conservative	ANK 1	Loss of cysteine in cat may affect local structure stability or redox sensitivity
136	R	V	L	Non-conservative	Non-conservative	ANK 1	Change-to-hydrophobic substitutions may alter ankyrin repeat packing or flexibility
140	Q	V	V	Non-conservative	Non-conservative	ANK 1	Polar-to-hydrophobic change may affect local folding or protein-protein interactions
141	D	E	E	Conservative	Conservative	ANK 1	Conservative acidic substitutions; likely maintains local charge balance
144	D	E	E	Conservative	Conservative	ANK 1	Conservative acidic substitution; unlikely to distrupt function
147	R	R	K	Identical	Conservative	ANK 2	Conservative basic substitution in cat; likely minimal functional impact
150	R	H	R	Conservative	Identical	ANK 2	Histidine substitution in human may introduce pH-sensitive modulation
151	G	D	G	Non-conservative	Identical	ANK 2	Introduction of acidic residue in human may affect local flexibility
152	L	E	L	Non-conservative	Identical	ANK 2	Hydrophobic-to-acidic change in human may alter structural packing
153	D	D	E	Identical	Conservative	ANK 2	Conservative acidic substitution in cat; likely preserves function
155	P	P	A	Identical	Non-conservative	ANK 2	Loss of proline in cat may affect local backbone rigidity
198	D	D	N	Identical	Non-conservative	ANK 3	Acidic-to-polar substitution in cat may influence temperature sensitivity
201	D	G	D	Non-conservative	Identical	ANK 3	Loss of acidic residue in human may alter local conformational flexibility
207	E	E	A	Identical	Non-conservative	ANK 3	Charge loss in cat may affect coupling between temperature sensing and gating
230	I	I	V	Identical	Conservative	ANK 4	Conservative hydrophobic substitution; unlikely to strongly affect channel behavior

231	T	A	T	Non-conservative	Identical	ANK 4	Loss of polar side chain in human may affect temperature-dependent conformational changes
233	V	L	L	Conservative	Conservative	ANK 4	Conservative hydrophobic substitution; likely preserves transmembrane packing
246	K	K	S	Identical	Non-conservative	ANK 4	Charge loss in cat may affect electrostatic interactions within the temperature-sensing domain
248	V	A	V	Conservative	Identical	ANK 4	Conservative hydrophobic substitution in human
284	N	H	N	Non-conservative	Identical	ANK 5	Introduction of histidine in human may confer pH-dependent modulation
292	Q	R	Q	Non-conservative	Identical	ANK 5	Charge gain in human may alter inter-helical interactions
407	I	T	I	Non-conservative	Identical	S1	Polar substitution in human may influence coupling between sensing and pore opening
439	Y	H	Y	Non-conservative	Identical	S2	Aromatic-to-polar substitution in human may affect helix stability of gating
468	D	A	A	Non-conservative	Non-conservative	S3	Loss of acidic residue in both human and cat may affect pore gating dynamics
480	S	G	G	Non-conservative	Non-conservative	S3	Increased flexibility in human and cat may influence channel opening kinetics
528	V	I	V	Conservative	Identical	S4-S5 linker	Conservative hydrophobic substitution in human; likely minor structural effect
